# Release Monitoring and Detection of Formulated Solid Nanoparticle–Conjugated Nicotine in Blood and Urine Using Electrochemical Technique

**DOI:** 10.1002/ansa.70018

**Published:** 2025-05-11

**Authors:** Blessing Wisdom Ike, Joshua C. Nwabuife, John Alake, Darko Kwabena Adu, Lungelo Miya, Ruchika Chauhan, Zondi Nate, Rajshekhar Karpoormath, Mbuso Faya

**Affiliations:** ^1^ Department of Pharmaceutical Chemistry College of Health Sciences University of KwaZulu‐Natal Durban South Africa; ^2^ Discipline of Pharmaceutical Sciences College of Health Sciences University of KwaZulu‐Natal Durban South Africa; ^3^ Department of Pharmacology University of the Free State Bloemfontein South Africa; ^4^ Department of Chemistry Cape Peninsula University of Technology Bellville South Africa

**Keywords:** electrochemical sensors | nanoformulation and drug release | nicotine monitoring | SUDs | World Anti‐Doping Agency (WADA)

## Abstract

Tobacco (nicotine) has been reported as one of the worst global public health pandemics in history, claiming about 8 million lives annually. According to the World Health Organisation (WHO), nicotine accounts for about 7 million deaths of firsthand users and over 1.3 million morbidities of secondhand users. Furthermore, smokeless tobacco products have been linked to more than 300 million morbidities, including chronic kidney illnesses. On the basis of this trend, a possible increase of over 100% in mortality rate and a state of emergency have been predicted from now till 2050. However, electrochemical analysis has demonstrated cost‐effective and easily synthesised sensors as a timely alternative for the rapid analysis and quantification of nicotine in diverse products. A carbon‐based silver sensor was fabricated and characterised by energy‐dispersive x‐ray (EDX) spectroscopy, Fourier transform infrared (FTIR) spectroscopy, scanning electron microscopy (SEM), direct light scattering (DLS), and x‐ray diffraction (XRD). Herein, we report the first electrochemical detection, release monitoring and quantification of conjugated nicotine. The sensor showed a significant sensitivity, specificity and discriminating power with a detection and quantification limit of 2.283 × 10^−9^ and 0.761 × 10^−8^ M, respectively. An average recovery rate of 96.26% was recorded. The applicability of the modified electrode was examined in human urine and serum. The research showed the potential of this method for monitoring doping and nicotine release, as well as for diagnostic and quality control purposes.

## 1 Introduction

According to the World Health Organisation (WHO), the tobacco epidemic stands as one of the most severe global public health crises of all time [1]. The use of tobacco products poses a global health risk due to the several chemicals found in tobacco, many of which have been established to be detrimental to human health [2, 3, 4, 5]. Apart from the recent estimated 8 million mortality figures reported by the WHO, chronic kidney disorder (CKD), lung and oropharyngeal cancers, chronic obstructive pulmonary disease, stroke and cardiovascular issues are among the leading morbidities and mortalities that are attributed to the use of tobacco products [2, 3, 4, 5]. Nicotine has been identified as the causative agent linked to these public health concerns in tobacco products. Nicotine is the psychoactive amphiphilic alkaloid with addictive, stimulating and anxiolytic effects present in tobacco leaves (*Nicotiana tabacum*) [6]. It makes up over 95% of the estimated total alkaloid content in cigars, cigarettes and flake tobacco, with concentrations ranging from 1 to 30 mg/g [7]. Pharmacologically, nicotine triggers a psychoactive effect on the central nervous system (CNS), thereby increasing dopamine levels and producing transient feelings of euphoria, alertness and concentration [8, 9]. Due to this transient effect, relapse vulnerability is created by the brain to desire more tobacco consumption, which in turn releases more dopamine and leads to addiction and long‐term nicotine cravings and usage [10, 11].

In an apparent attempt to aid smokers in quitting or managing addictions, some smokeless nicotine products, including chewing gum, snuff, snus and pouches, are currently being manufactured by the tobacco industry as nicotine replacement therapy (NRT). Meanwhile, several questions and concerns have been debated on the safety of some of these products because they may still contain the psychoactive ingredient [4, 6, 11, 12]. There is a recommended daily nicotine intake of between 30 and 60 mg (this might vary from person to person) and regulatory measures. Meanwhile, the proliferation of various nicotine products (both fast‐ and sustained‐release nicotine products) may have now added layers of complexity to the already existing global health challenge and analytical technique (thereby overstretching the capacities of conventional instruments) [3, 5]. Apart from these direct health implications of nicotine usage, other ripple effects of nicotine consumption include economic, crime and environmental pollution [13, 14, 15, 16, 17, 18, 19]. Hence, it becomes imperative and of high priority to protect consumers and the environment and ensure that producers follow the rules regarding the amount of nicotine in these products; thus, it is critical to develop techniques that can easily and rapidly detect, monitor and quantify nicotine concentrations in users and these varieties of products, especially in various domains like sports, crime scenes, confiscated products, immigration points and borders.

Research focused on detecting and quantifying nicotine has a long history dating back to the early 20th century [20]. Initially, researchers focused on understanding the effects of nicotine on human health, particularly about smoking and tobacco use [21]. As concerns about nicotine addiction and related health issues grew, scientists began exploring ways to detect and measure nicotine levels in different substances and formulations. Several conventional methods, such as spectroscopy, micro/macroscopic chromatography and electrophoresis, have been used to detect nicotine [22, 23, 24, 25, 26]. Meanwhile, chromatographic and spectroscopic methods remain the most widely used nicotine analysis methods. Electrochemical techniques have previously been used for nicotine analysis; however, most of these analyses focused more on fast‐release nicotine, with the paucity of research on slow‐release products currently flooding public spaces indiscriminately [23, 26]. Thus, a rapid, environmentally conscious, cost‐effective and simple analysis technique is critically necessary for these product varieties. Although spectroscopic and chromatographic techniques can identify and measure nicotine, unfortunately, some drawbacks, such as time consumption, expensive price tags, onsite nondeployable, extensive use of bulk and environmentally unconscious solvents and high operational skills, remain challenging [27, 28]. In addition to this list are sample pretreatment challenges and method developments, especially with the chromatography method.

On the other hand, UV–Vis, unlike the chromatographic technique, could be one of the most affected conventional instruments in terms of analytical power. Significant limitations such as discriminating ability, selectivity, sensitivity and accuracy can be an issue with the UV–Vis [29]. For instance, hydrating structures like dendrimers, liposomes, nano‐emulsions, micelles and nanoparticles are usually used as nanocarriers to formulate several sustained‐release nicotine products because of their desired tailored activities [30, 31, 32]. However, the pH of such an aqueous solution used for the formulation could present false positive results when measured with the spectrophotometric method. Hence, the sum of the spectra of several species may equate to spectra obtained for the test analyte [29]. Moreover, some chromophore materials with overlapping bands can absorb light at the same wavelength as the target analyte; this could also lead to less sample discrimination and interference, poor sensitivity and probable false positive results [29]. These underscore the urgent need for a more comprehensive intervention. As such, addressing the nicotine epidemic requires a multifaceted approach encompassing stringent regulations, more public awareness campaigns and, above all, simple and robust analytical methods like electrochemical sensors.

Significant analytical impacts like improved sensitivity, selectivity, higher accuracy, simplicity, affordability, reliability, onsite deployability, user‐friendly interface and minimal solvent usage are all attributes of electrochemical sensors. Even though some electrochemical sensors have been developed to detect nicotine, issues like sensitivity and the over‐potential of nicotine on the electrode surface call for more alternative platforms to overcome this issue [33]. To solve this problem, scientists have focused on developing nanomaterials for tailored functional electrochemical kinetics. This is because of their affordability, stability and suitable analytical responses [11, 12]. Carbon‐based materials, dendrimers, polymers, composite materials and nanoparticles, amongst others, have been explored for several Faradaic processes due to their redox potentials, stability, befitting nano ranges, abundance, affordability and simple synthesis [30, 34, 35]. Nanoparticles have been among the leading materials in electrochemical studies [31, 36, 37, 38, 39, 40, 41, 42, 43]. Though stability may have been one of the concerns for silver nanoparticles, researchers have reported that stabilising and anchoring agents, like lemon peel extracts and charcoal, amongst others, can address this challenge in a Faradaic process without the use of chemical binders [44, 45, 46, 47]. Hence, considering the availability of charcoal and lemon peel biomass in every facet of life, functional, stable carbon‐anchored silver prevailing nanoparticles (C‐AgNPs) were sourced and repurposed in an eco‐friendly way and developed into a functional sensor for the detection of regular and formulated slow‐release nicotine in solid nanoparticles conjugate.

This research aims to develop a reliable and efficient electrochemical method for detecting, monitoring the release and quantifying nicotine in various products and pharmaceuticals for potential applications in several domains, such as sports, immigration, border points and point‐of‐care. Therefore, the scientific findings in this study may present an alternative electrochemical method for detecting and quantitatively analysing diverse nicotine products (both fast and sustained release).

## 2 Experimental Procedures

### 2.1 Materials and Instrumentation

Analytical grades (99%) of stearic acid, cholesterol, chloroform, sodium hydroxide, potassium phosphate, potassium hydrogen phosphate, ethanol, potassium ferrocyanide, potassium ferricyanide, sheep blood and nicotine were all acquired from Sigma‐Aldrich South Africa (Pty) Limited. A lemon was purchased from a local supermarket in Durban, South Africa. A dead tree branch was collected from a deforested tree at the Westville Campus of the University of KwaZulu‐Natal. Deionised water (resistivity ≥ 18 MΩ cm and conductivity ≤ 0.056 µS/cm) was used for all electrochemical solution preparation and washing of electrodes. Water used during the formulation study was distilled in the laboratory using the Milli‐*Q* water purification system (Millipore Corp., USA).

MSH 140 Ultra Turrax Homogeniser (Boeco, Germany), Rotatory evaporator (EINS Sci E‐RE‐V), STUART Orbital Incubator SI500, Zetasizer Nano ZS90 (Malvern Instruments Ltd., UK), Amicon Ultra‐4 filter centrifuge (Millipore Corp., USA), Milli‐*Q* Water Purification System (Millipore Corp., USA). UV spectrophotometer Model: UV‐1800, Shimadzu, South Africa. Field emission scanning microscopy (FE‐SEM) (ZEISS Ultra Plus, Germany) and transmission electron microscopy (TEM) (JEOL 101, USA), energy dispersive x‐ray spectroscopy (EDX), powder x‐ray diffraction (PXRD) Goniometer MiniFlex 300/600 1D scan diffractometer at 40 kV, 15 mA x‐ray generator supplied with monochromatic Cu *K*_α_ radiation. Fourier transform infrared (FTIR) spectroscopy (Bruker Alpha‐PATR‐FTIR, Germany), CH1660E electrochemical workstation (CH instrument, USA) with a three‐electrode system; Ag/AgCl (reference electrode), GCE (working electrode 3.0 mm) and platinum wire (counter electrode) instruments were all deployed for every related analysis and measurement in this study.

### 2.2 Preparation and Synthesis

#### 2.2.1 Preparation of Charcoal

A 15 cm branch of a deforested random tree was collected from the University of KwaZulu‐Natal Westville campus. It was then debarked, cleaned with water and left in the sun for 5 days. The wood was burnt over an open flame until the biomass became solid charcoal. After cooling with deionised water, the resulting charcoal was sun‐dried for 48 h before crushing. The black biomass powder of 3 g was weighed and reserved for further use.

#### 2.2.2 Preparation of Lemon Peel Crude Extract

Prepping the lemon peel crude extract began with washing an average‐sized yellow lemon with water, after which the peel was obtained and sun‐dried for 5 days. The dried peel was milled, and 5 g of the powdered peel was weighed and soaked in 150 mL of ethanol for 24 h (ideal for both polar and non‐polar extraction). The mixture was filtered thrice with a 47 mm pore size Whatman filter paper to remove solid particles, and the resulting dark brown filtrate was covered and stored at room temperature for 24 h before use.

#### 2.2.3 Synthesis of Dual‐Functionalised Carbon‐Complexed Silver Nanoparticles (C‐AgNPs)

The nanoparticle was prepared by aqueous colloidal synthesis, following the method established in a previous study with some modifications [48]. At first, 2.59 g of charcoal and lemon peel extract (5 mL) were added to 100 mL of deionised water. The dark brown solution was adjusted to a basic pH of 12 using 1 M NaOH. The solution was then bubbled with nitrogen for 30 min and labelled Solution A. In a separate flask B, 95 mL of deionised water containing 1.58 g of silver nitrate and 5 mL of lemon peel extract (a dual functionalisation aimed at reducing silver nitrate and stabilising the resulting silver nanoparticles) was prepared. This solution was stirred for 30 min, and then 50 mL of solution B was rapidly transferred to solution A. The reacting components were stirred vigorously and refluxed at 100°C for 24 h. After 24 h, the reaction was quenched by rinsing the solution with cold water. The resulting dark brown solution was washed three times for 10 min, first with propan‐2‐ol and then with deionised water to remove any excess precursors that might have formed. Deionised water was used to remove possible remaining precursors after each centrifuge ran at 4500 rpm. The residue was oven‐dried at 80°C for 12 h and stored at room temperature for later use.

#### 2.2.4 Fabrication of the Electrochemical Sensor

First, the working carbon electrode was polished in a micro‐alumina slurry of 0.05 µm and rinsed with deionised water to rid the electrode surface of any adsorbed alumina. After that, cyclic voltammetry (CV) was used to confirm the purity of the electrode using 2.5 mM [Fe(CN)_6_]^3−^/^4−^ redox pair in 0.1 M KCl electrolyte solution at a scan rate of 0.1 V/s and a potential range of −0.4 to 0.6 V and a peak current separation of 1 V was achieved. Then, the carbon electrode was coated with 5 µL of the C‐AgNPs and allowed to dry under the infrared lamp for 10 min. The modified electrode was used for the electrochemical measurement throughout this study. For comparison, the bare (GCE) and modified electrodes were characterised using electrochemical impedance spectroscopy (EIS), CV and differential pulse voltammetry (DPV).

#### 2.2.5 Formulation of Nicotine Solid Nanoparticles (N‐SLNPs)

The nicotine‐encapsulated SLN was prepared using a previously reported method with some modifications [26]. An organic solvent (chloroform) was used to dissolve a mixture of stearic acid and cholesterol with a ratio of 1:2 and 0.5 mL of linolenic acid. The mixture was then dispersed into 10 mL of phosphate buffer (PB) (pH 7.4) and homogenised for 90 s at 6000 UpM. The organic solvent was then evaporated using a rotatory evaporator. Nicotine with a concentration of 250 µg/mL of the final formulation volume was added to the resulting mixture, and the final volume of the formulation was made up to 20 mL. Lastly, the mixture was further homogenised for 3 min to form N‐SLNP. The prepared N‐SLNP was then preserved at 4°C for further use. The blank formulation (BF) was similarly prepared, but this time, without adding nicotine.

#### 2.2.6 Preparation of Urine and Blood

The practical applicability of the modified electrode was examined in urine (obtained from healthy laboratory personnel) and sheep's blood. The urine and whole blood were first centrifuged at 4500 rpm, and the supernatants were collected and diluted 100 times in pH 7.4 PB to reduce the matrix effect. The diluted urine and serum samples were spiked with increasing formulated nicotine concentrations (10–100 µM) for electrochemical detection using the modified electrode. The practical applicability of the modified electrode was also examined in urine and serum.

#### 2.2.7 Electrochemical Detection N‐SLNP With the Fabricated Electrochemical Sensor

In PB with varying pH levels (3.0–8.0) within a potential window of 0–1.6 V, the lead pH (7.4) based on the optimal electrochemical response and its impact on the interaction between C‐AgNPs/GCE and N‐SLNP was evaluated using DPV. The optimal pH for more testing was determined to be 7.4. The results section includes the data and discussion from the pH study. The nature of the redox reaction was subsequently investigated using CV at a scan rate of 0.1 V using the optimal pH and the same potential window. Subsequently, the scan rate was adjusted between 20 and 120 mV to examine its impact.

#### 2.2.8 Determination of In Vitro Drug Release of N‐SLNP Formulation

To determine the drug release profile mechanism of prepared N‐SLNP, an in vitro release analysis of the entrapped drug was conducted according to previously reported methods [26, 49]. The nicotine in N‐SLNP was released through diffusion with the help of a dialysis bag with pore sizes ranging from 8000 to 14,400 Da. About 2 mL of bare nicotine, BF (S‐SLNP) and N‐SLNP formulations were placed into each dialysis bag. The dialysis bags were each placed in a receiver chamber containing 40 mL PBS (pH 7.4) using a shaking incubator at 100 rpm at 37°C. At intervals of 0.5, 1, 2, 3, 4, 5, 6, 7, 8, 12, 24 and 48 h precisely, 3 mL was extracted from the receiver chamber and replaced using the same amount of incubated PBS (pH 7.4) during the sampling process to maintain a constant volume in the compartment.

## 3 Results and Discussion

### 3.1 Characterisation of Materials

**FIGURE 1:**
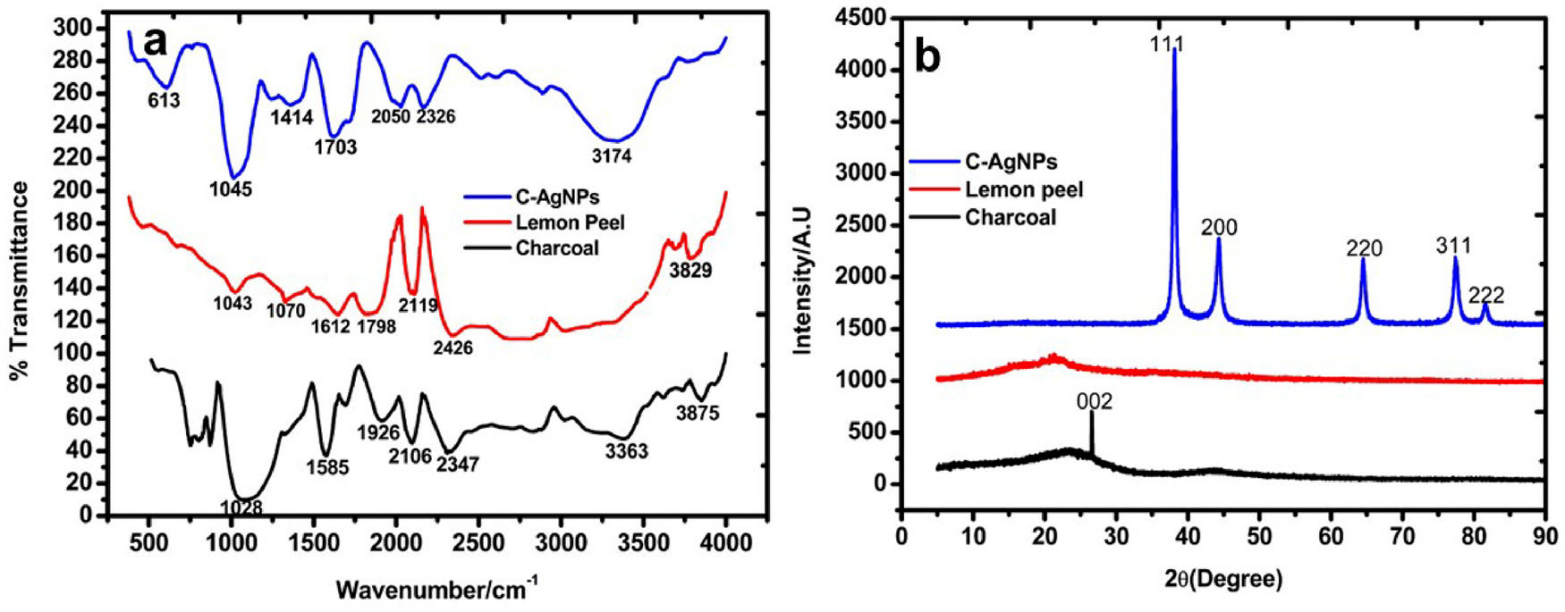
(a) FTIR and (b) x‐ray diffraction analysis of the components and the C‐AgNP composite.

FTIR was used to characterise the components of the nanomaterial sensor. Starting with the charcoal (Figure 1a), a characteristic broad and strong O─H stretching starting from 3875 to 3363 cm^−1^ was observed. This is reported to represent the O─H stretching vibration of hydrocarbons [46, 50]. The peaks at 2347 cm^−1^ can be linked to the atmospheric absorbed carbon dioxide [50]. The peaks of 1414–1926 cm^−1^ are characteristics of the hydroxyl group and the absorbance of water [50, 51]. The aliphatic CH deformation is predicted in the band at 1028 cm^−1^ [51, 52]. In addition (Figure 1a), the analysis of the lemon peel was carried out, and the FTIR significant peaks consistent with the components of limonene and monoterpene were seen at various distinguishable band locations, starting with the broadening of O─H stretching (R─C(O)─OH) around 3829 cm^−1^ are attributed to carboxylic acid and other intermolecular bonded strong O─H stretching [46, 53]. The sensor revealed the alkane functional groups’ C─H stretch peaks at 2426 and 2119 cm^−1^ were recorded as previously for carbonaceous wood charcoal [53]. Moreover, the C═O stretching (C(O)─OH) at 1798 cm^−1^ to 1612 has been reported for strong alkene monosubstituted stretching, and the C─OH stretching at 1070–1043 cm^−1^ is depicted for saturated alkane medium [46, 53]. Bands resulting from O─H stretching (about 3174 cm^−1^), aldehydic C─H stretching (2326 cm^−1^), C═O group (2050 cm^−1^) and C─C (1414 cm^−1^) stretching, and the peak at 613 cm^−1^ can be attributed to medium alkene (C═C) trisubstituted bond [46]. These FTIR spectra are in agreement with the literature about the successful formation of C‐AgNPs [37].

The XRD analysis (Figure 1b) was conducted to confirm the XRD of the components and nanoparticle's pattern. One of the components, the charcoal's XRD pattern, shows a prominent amorphic planner peak 0 0 2 with a Crystallography Open Database Card Number 9011577; these data agree with the previously reported graphitic carbon materials of charcoal origin [51, 52]. Furthermore, the XRD pattern of the milled lemon peel shows some amorphous characteristic peaks in conformity with previously reported data [54]. The nanomaterial sensor revealed 5 distinct diffraction points at 2*θ* peaks at 39, 44, 64, 44 and 81, which are indexed to 111,200, 220, 311 and 222 with COD: 9008459 and can be linked to the planes of the face‐centred cubic (fcc) structure and are consistent with the fcc and crystalline nature of AgNPs [55, 56].

#### 3.1.1 Microscopic Characterisation

**FIGURE 2:**
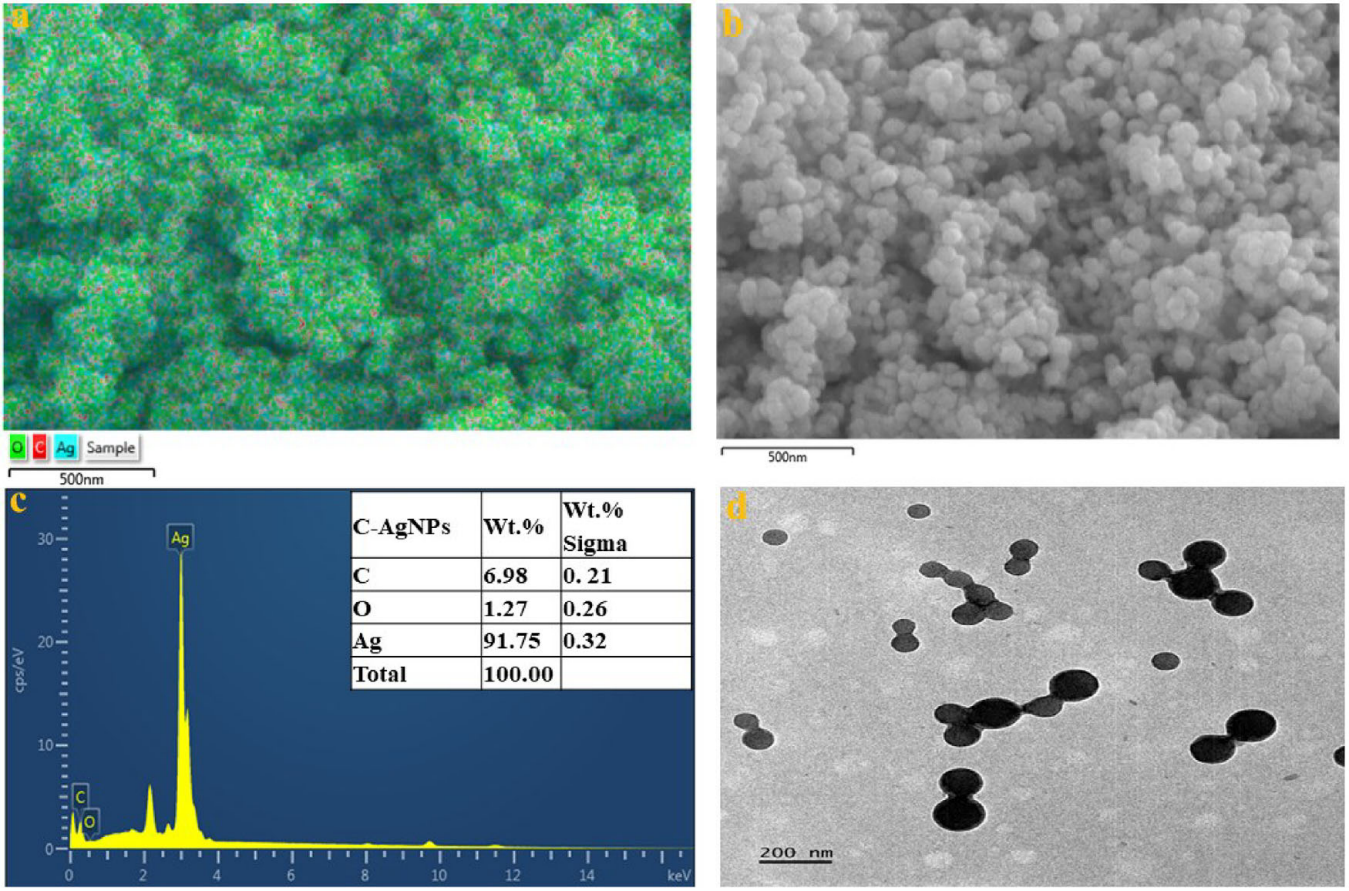
(a–c) EDX mapping and EDX spectra and (d) TEM of the C‐AgNPs.

At first, the charcoal and lemon peel's elemental composition, EDX mapping and topological morphology were analysed using SEM and TEM, respectively, as revealed in Figure S1. Then, the anticipated elemental composition of the synthesised C‐AgNPs functionalised biomass contained 91.75% silver, 6.98% carbon and 1.27% oxygen (Figure 2a,c), which points to the successful formation of an intended Ag current material. Meanwhile, these elemental compositions are anticipated as more silver may be scanned in a topological view or angle, as the enlarged anchoring porous carbon surface is activated for lots of silver adsorption due to the functional effect of the lemon peel extract. SEM scanning was conducted further to confirm the topological or surface morphology of the materials. The Ag‐rich material surface at different magnifications revealed a spherical form of C‐AgNPs with a broad size distribution. Furthermore, TEM scanning was carried out to get more details about the inner or individual particles, as depicted in Figure 2d; the C‐AgNPs revealed a non‐agglutinated size distribution and approximately spherical shapes. The results confirm the successful synthesis of the anticipated well‐silver‐captured nanoparticles [57].

**FIGURE 3:**
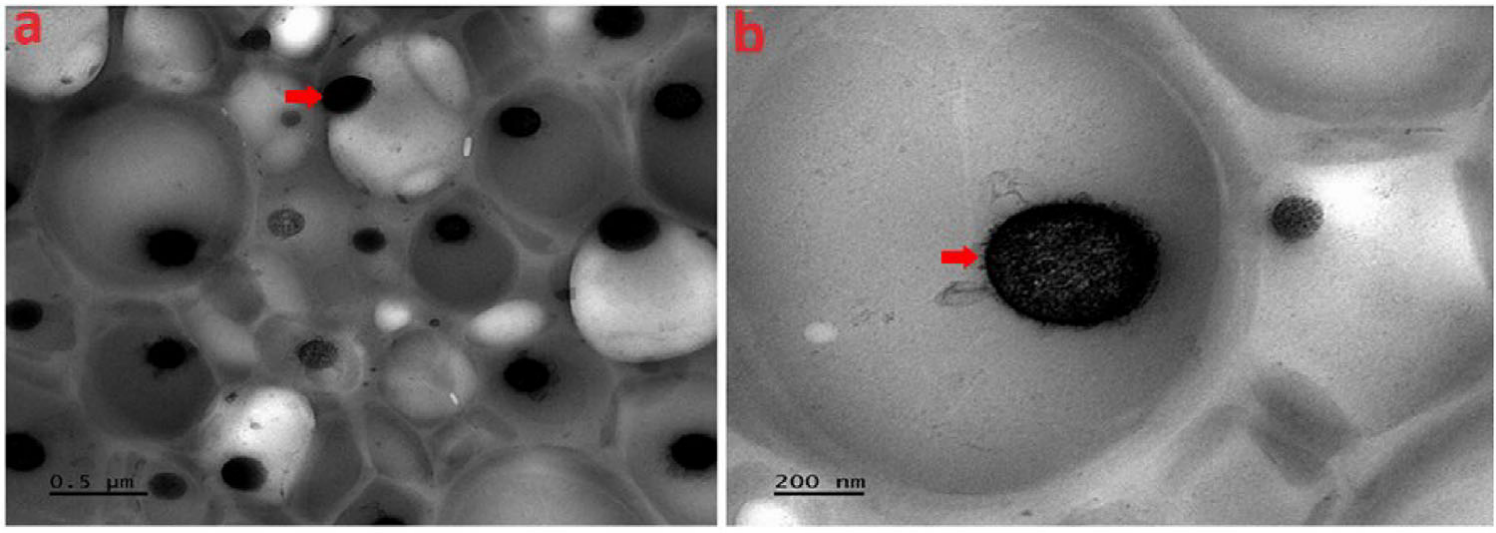
(a and b) TEM images of N‐SLNP.

Furthermore, the TEM scanning of the N‐SLNP was also conducted to capture the surface morphology of the entrapped nicotine in the nanoformulation, as indicated by the red arrow in Figure 3a. A solid‐phase nicotine entrapped, non‐agglutinated homogeneous particle distribution was recorded. Obvious spherical shapes were observed. These results also confirm the N‐SLNP synthesis's effectiveness via the homogenisation technique.

#### 3.1.2 DLS Particle Size and Charge Characterisation of N‐SLNP

**FIGURE 4:**
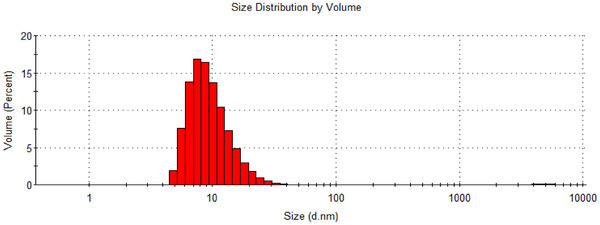
DLS infographics of the size distribution of the N‐SLNP.

Because the diffusion coefficient (*D*) of the particles is inversely proportional to the size (dp hydrodynamic diameter [DH]) of the particles, DH, PDI and *ζ* of the N‐SLNP were carried out by dynamic light scattering (DLS) using a Zetasizer. Preliminary studies were performed to obtain the optimal formulation used for the study. The optimised formulation demonstrated DH, PDI and ζ of 19.85 ± 2.091 nm, 0.22 ± 0.02 and −17.4 ± 0.25 mV, respectively (Figure 4). The DLS particle size data are well within the nano‐size, confirming the formation of nano‐sized nicotine.

The various characterisation techniques project suitable shapes, elemental compositions, good formation, sizes and polydispersity of the C‐AgNPs and the interacting SLNP analyte. These favourable characteristics are expected to improve the electrochemical interaction. The high surface area–to‐volume ratio of both C‐AgNPs and SLNP may lead to an increase in the surface available for binding, catalysis or reaction with surrounding materials, which potentially improves the interaction and, subsequently, the detection [39, 58, 59, 60].

### 3.2 Electrochemical Characterisation of the Fabricated Sensor

**FIGURE 5:**
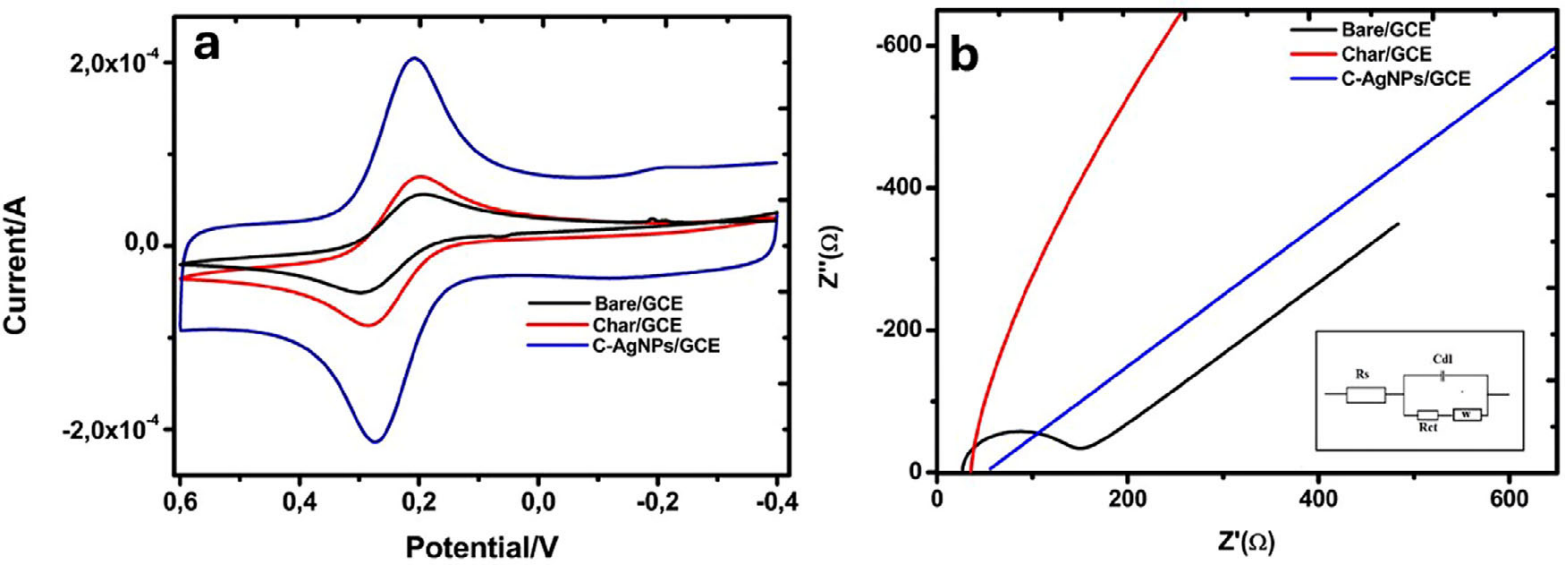
(a) CV of various electrodes and (b) Nyquist plots from the EIS of the electrodes. *Note*: Inset is Randel's equivalent circuit.

Voltammetric techniques are an electrochemical analysis used to study the relationship between voltage and current in an electrochemical cell. These techniques involve applying a potential difference between two electrodes and measuring the resulting current. However, these techniques may sometimes be limited by interference from impurities, electrode fouling, sensitivity to experimental conditions and matrix effects. However, the tailored tunability and functionalities of nanomaterial modifiers, such as carbon, metal and nanocomposite‐based materials, for sensor applications have proved helpful in resolving these challenges [61, 62]. Alternatively, chemometric methods of second‐order data analysis (multivariate curve resolution‐alternating least squares [MCR‐ALS]) have been reported for resolving the issues of overlapping peaks, which may result from voltammetric techniques [63]. To confirm the electrocatalytic characteristics of the fabricated sensor and the improved catalytic effect of the modifiers and the composite in resolving this concern, CV (Figure 5a) and EIS (Figure 5b) measurements were carried out. The anodic peak current (*I*_Pa_) of 5.798 × 10^−5^ A and the cathodic peak current (*I*_Pc_) of 5.475 × 10^−5^ A were obtained from the Glassy Carbon Electrode (GCE) CV analysis with a corresponding potential (Ep) of 0.306 and 0.199 V, respectively. After the GCE surface was modified using charcoal (Char/GCE), the *I*_Pa_ and *I*_Pc_ increased to 8.707 × 10^−5^ A and 8.349 × 10^−5^ A with Ep values of 0.285 and 0.197 V, respectively. With the C‐AgNPs/GCE, an even more significant increase in the *I*_Pa_ and *I*_Pc_ to 1.814 × 10^−4^ A and 1.707 × 10^−4^ A with a corresponding Ep of 0.273 and 0.207 V was observed. The peak increment trend following modifications shows that the coating materials have improved the GCE's conductivity (Figure 5a). According to the CV data, the nanoparticles were the best material for this investigation, showing a more beneficial synergistic electrochemical output than the separate components.

In addition, EIS analysis was performed to ascertain the electron transfer and resistance rate. In an electric circuit or component, the combination of reactance and ohmic resistance produces effective resistance to alternating current. Lower impedance to electron transport is achieved with a low *R*_ct_ value. The *R*_ct_ for the bare GCE was 156.9 Ω, Char/GCE was 29.55 Ω and the C‐AgNPs/GCE was 0.001 Ω. The EIS is shown graphically in Figure 5b. This Nyquist plot (Figure 5b) shows the relationship between actual resistance (*Z*′) and imaginary resistance (*Z*″) fitted using Randel's equivalent circuit (Figure 5b, inset). The charge transfer resistance (*R*_ct_) is directly proportional to the semi‐circle's size in a Nyquist plot. It is evident from Figure 4b that the modified electrodes do not exhibit any significant semi‐circles or radii, as the bare GCE does; hence, the electron transfer rate, according to the results, shows to be the fastest. This observation agrees with the CV analysis where the combined material, C‐AgNPs, showed the highest, most significant current value and the lowest peak separation Δ*E* observed in the study. This result further supports the data gathered from the CV and DLS study, demonstrating through experimentation that miniature sizes of particles are beneficial for increased conductivity and catalytic responsiveness [39]. A summary of the CV and EIS data is presented in Table 1.

**TABLE 1:** GCE and modified electrodes cyclic voltammetry (CV) and electrochemical impedance spectroscopy (EIS) measurement and values.

Materials	CV		EIS		*C*_dl_ (F/g)	*W* (Ω/s^1/2^)
	*I*_pa_/*I*_pc_	Δ*E* (mV)	*R*1 (Ω)	*R*_ct_ (Ω)		
**Bare/GCE**	1.02	107	24.87	156.9	9.416 × 10^−7^	0.00004213
**Char/GCE**	1.04	88	26.85	29.55	4.415 × 10^−6^	0.00008082
**C‐AgNPs/GCE**	1.06	66	50.74	0.001	1.010 × 10^−5^	0.00001962

Abbreviations: GCE, Glassy Carbon Electrode; *R*, resistance; *R*_ct_, charge transfer resistance; *C*_dl_, double‐layer capacitance; *W*, Warburg impedance.

Studies have shown improved electron transfer rates of nanoparticles; silver and carbonaceous materials have been reported among the leading materials for such Faradaic purposes [30, 46].

#### 3.2.1 Surface Area of the Modified Electrode (C‐AgNPs/GCE)

CV measurements were performed using C‐AgNPs/GCE and bare GCE at a scan rate of 10–500 mV/s, and a potential window of −0.4 to 0.6 V. To determine the effective surface area of the electrodes, a graph of anodic current against the square root of the scan rate is obtained Figure S2. The slope obtained from the linear plot was used to calculate the effective surface area of the electrode using the Randles–Sevcik formula, expressed in the following equation:

(1) $$I_{P}=2.69\times 10^{5}\;\left(\sqrt{D}\right)\left(A\right){\left(\surd N\right)}^{3}\left(\sqrt{V}\right)\left(\mathrm{Co}\right)$$

where the *D* diffusion coefficient of the K_3_[Fe (CN)_6_] is 7.60 × 10^−6^ cm^2^/s, *I*_p_ is peak current (A). *V* is the scan rate in V/s, Co is the concentration in mol/cm^3^ of K_3_[Fe (CN)_6_], *A* is the electrode's surface area in cm^2^, and *n* = 1 (the number of electrons transferred). The calculated active surface areas for the bare, charcoal and C‐AgNP‐modified electrodes were determined to be 0.032, 0.107 and 0.187 cm^2^, respectively, and were denoted by the value of *A*. The conducting properties of metal (Ag) [56, 60, 64], in synergy with carbonaceous or graphitic [65, 66] composites, can be attributed to the electrode's increased electroactive surface area and sensitivity.

#### 3.2.2 Electrochemical Behaviour and Performance of Bare/NIC and N‐SLNP Towards C‐AgNPs/GCE

In this preliminary investigation, the sensor's specificity was investigated within nicotine's window potential, wherein the bare electrode was first utilised to test the buffer, as depicted in Figure S3. After that, C‐AgNPs/GCE was used to detect 0.1 M standard (fast‐release nicotine Bare/NIC). The delivery system (BF) was also tested using C‐AgNPs/GCE to check for potential interference within the nicotine oxidation window. Lastly, C‐AgNPs/GCE was used to detect 0.1 mM N‐SLN concentration in PB pH 7.4. This confirms the sensor's specificity and selectivity for nicotine in every form (sustained and fast release). This suggests a discriminating rate of the electrochemical sensors, considering the absence of any observable peak response in the blank PB and BF (Figure S1b). Hence, no physical or electrochemical interference was observed from the bare formulation or blank PB. However, no discernible peak was found with the bare/GCE in 0.1 M formulated and unformulated nicotine. Instead, a notable peak was seen at 0.98 V with a current of 2.889 × 10^−4^ A after modification with C‐AgNPs/GCE (Figure S3). This result validates the sensor's successful penetration into the nanosystem and optimal interaction with the target analyte; this suggests the selectivity and catalytic impact of the nanomaterial deposited on the electrode surface.

The pH study significantly affects the sensitivity of an electrochemical system, as it plays a crucial role in biological and chemical interactions or reactions. PB medium of pH 7.4 of 0.1 M has been severally reported as the optimal condition for nicotine detection [67, 68, 69]. Generally, pH 7.4 has been reported to show a balanced physiological relevance, stability and biocompatibility in biological systems [7, 69]. The formulation and characterisation studies revealed an optimal stable environment and better responsiveness at pH 7.4; hence, it was ideally chosen for all further analyses. However, to further confirm this, one pH below (7.0) and above (8.0) pH 7.4 were selected and tested, and the maximum Faradaic current efficiency (Faraday efficiency [FE]) was confirmed at pH 7.4. As represented in Figure S3b, the pH below or above 7.4 produced significantly lower currents. Scientifically, protons (H^+^) are abundant in acidic media; hence, the clouding of H^+^ in acidic media may result in less FE, interaction and sensitivity of some materials [70]. In essence, lower FE and peak currents may be due to the hydrogen evolution process (HER) [70]. So, for optimal interaction, such material must transit into a low proton concentration (neutral to basic) zone [70]. To transit into a neutral or basic region, higher currents (optimal peaks) are required to draw/pull the H^+^ ions at lower concentrations towards the electrode surface [70], which results in higher/optimal peak currents or FE.

### 3.3 Analytical Calibration Measurement

The calibration curve of the standard nicotine, established between the current and concentration, was measured using DPV to determine the linear regression measure, and this yields a model described by the mathematical expression shown in the following equation:

(2) $$\;I_{P}=1.330\times 10^{-3}\;\left(\mathrm{BNIC}\right)-4.234\times 10^{-6}\left(R^{2}=0.997\;\right)$$

**FIGURE 6:**
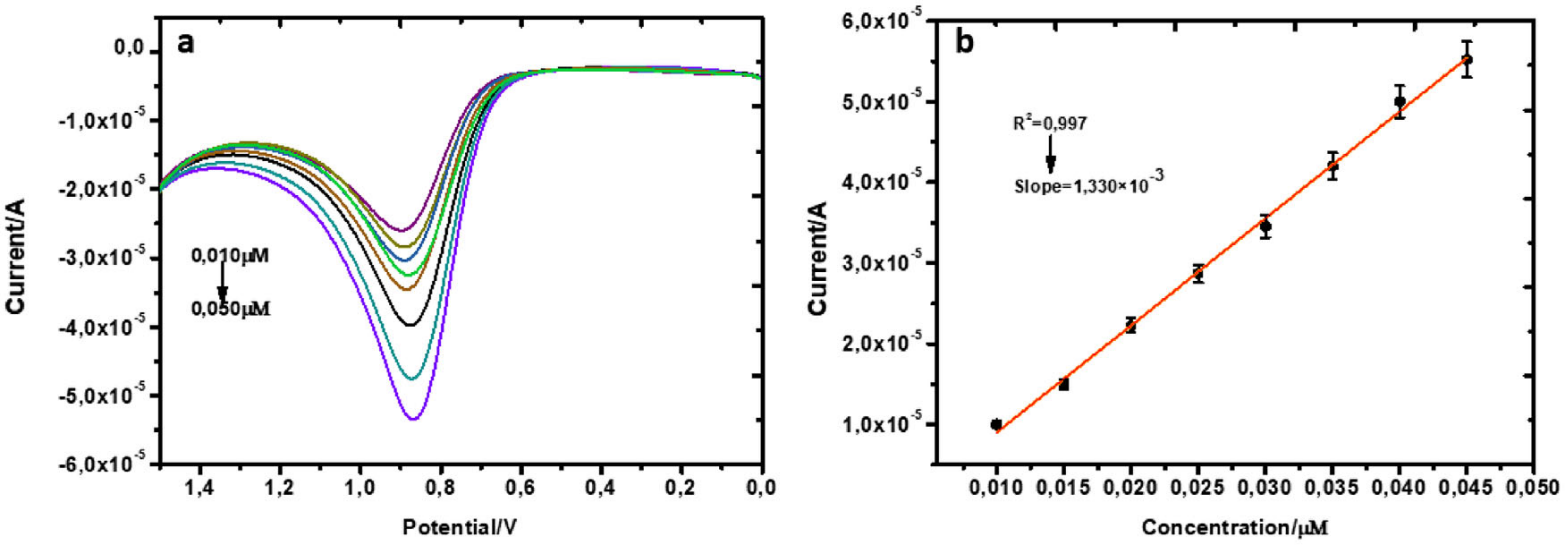
(a and b) The DP voltammogram and the calibration curve of the bare/NIC.

The analytical performances were calculated to determine the lowest and highest amounts of the analyte that the sensor can detect. Hence, the limit of detection and quantification was calculated using the equations, respectively:

(3) $$\mathrm{LOD}=\frac{3S}{M}\;$$

(4) $$\mathrm{LOQ}=10\frac{S}{M}$$

where *S* is the bare electrode standard deviation (= 1.012 × 10^−12^) in the blank PB, and *M* is the slope of the linear plot showing the peak current versus concentration (Equation 2). Using the extracted slope from the electrochemical method, the limit of detection and quantification was calculated to be 2.283 × 10^−9^ and 0.761 × 10^−8^ M, respectively. The calculated LOD appears to be the lowest and best‐performing silver‐based sensor according to the comparative LOD analysis reported in Table 2. The system's capacity to proportionately sustain a linear relationship between increasing concentration and current (*I*_p_) indicates the sensitivity and robustness of the sensor. This also may have impacted the significant detection limit of the second‐best performing sensor reported by M. Abd‐Elsabour et al. in Table 2. Thus, the outperformance of the C‐AgNPs in this study could have been much improved by a synergistic effect of the highly conductive carbon and silver hybrid material, which could ultimately facilitate rapid electron transfer observed in the electrochemical characterisation (Figure 6).

**TABLE 2:** Comparison of this electrode with other reported electrodes for nicotine sensing.

Technique	Electrodes	Linear range (µM)	LOD (µM)	Target	Refs.
DPV	RGO/DPA/PGE	31–1900	7.6 × 10^−6^	Fr‐Nic	[72]
CV	FC‐MWCNT/SPCE	60–1000	4.25 × 10^−6^	Fr‐Nic	[73]
DPV	bAuNPs/SPE	10–2000	2.4 × 10^−6^	Fr‐Nic	[74]
SWV	Fe doped MgNi_2_O_3_	50–6000	9.8 × 10^−6^	Fr‐Nic	[75]
DPV	MWCNT‐G/GCE	2–600	5.2 × 10^−7^	Fr‐Nic	[76]
CV	Ag‐NPs/GCE	2.5–105	1.4 × 10^−7^	Fr‐Nic	[56]
SWV	p‐ABSA grafted SPCE	0.5–300	3.5 × 10^−7^	Fr‐Nic	[77]
CV/CA	ACMCPE	4.0–320.0	7.6 × 10^−6^	Fr‐Nic	[78]
CV	AOX‐PAN/GCE	1.00–1000	4.5 × 10^−5^	Fr‐Nic	[79]
DPV	CNF‐PAMAM GCE	2.6–105	2.6 × 10^−6^	Fr‐Nic	[80]
CV	Mn/Cu NPs/CPE	10–6000	1.2 × 10^−6^	Fr‐Nic	[81]
DPV	CNF‐PAMAM GCE	0.4815–15.41	2.7 × 10^−5^	Fr‐Nic	[80]
CV	Ag‐NPs/GCE	2.5–105	1.35 × 10^−4^	Fr‐Nic	[56]
DPV	GO/Nq/GCE	6.5–245	12.7 × 10^−9^	Fr‐Nic	[82]
DPV	C‐AgNPs/GCE	0.02–0.095	2.283 × 10^−9^	Fr & Sr‐Nic	This study

Abbreviations: CA, chronoamperometry; CV, cyclic voltammetry; DPV, differential pulse voltammetry; Fr NIC, fast‐release nicotine; SWV, square wave voltammetry; Sr, sustained‐release nicotine.

### 3.4 Controlled and Uncontrolled Nicotine Release Monitoring

**FIGURE 7:**
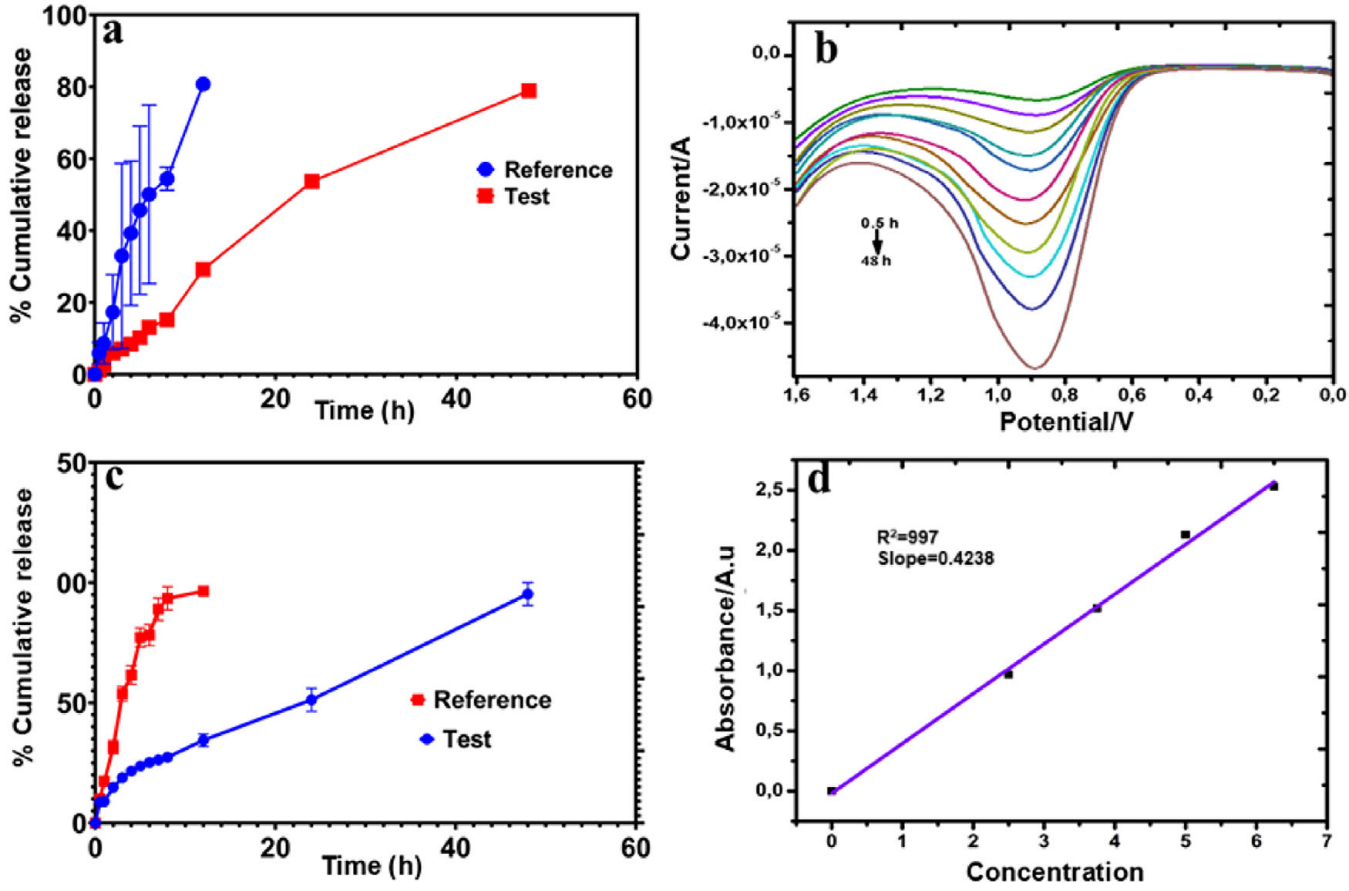
(a and b) The Bare/Nic and N‐SLNP electrochemical release profiles for the respective h/the DPV voltammogram; (c) UV–Vis the release profile of bare/NIC and N‐SLNP for the respective h; (d) the calibration plot for the UV–Vis spectroscopy.

Drug release profiling is formally presented as a cumulative percentage release curve to predict the quantity of drug accumulation in the body system over time. The in vitro release profile of the test and reference drug was investigated using the dialysis bag method in PB at pH 7.4 at 37°C for 48 h. Thereafter, at 0.5, 1, 2, 3, 4, 5, 6, 8, 12, 24 and 48 h, 3 (1 × 3) mL of the test and reference drug were drawn from the receiver solution into three different release vials and instantly replaced with an equivalent 3 mL PB to maintain the 40 mL total volume throughout the study. The released amount of the uncontrolled and controlled nicotine was monitored by electrochemical and UV–Vis techniques. Comparisons between the controlled and uncontrolled nicotine via both techniques were profiled and estimated. The experiment was performed in triplicate. Comparatively, as time increases, the current and absorbance also increase for both techniques; this expected increase suggests the anticipated accumulation of nicotine concentrations released in the system over time (Figure 7b). The release pattern or profile of the bare (uncontrolled) drug was similarly shorter than expected and lasted for only 12 h for both methods (Figure 7a,b). The controlled‐release drug was successfully monitored with a similar sustained‐release pattern for a further 48 h. Interestingly, the electrochemical method shows a consistent peak for the respective hours. In addition, the % cumulative fractions released for each hour were estimated using an Excel‐dd solver. In contrast, the UV–Vis technique showed a higher percentage released at each interval, with an accumulation of 96% and 95% at 12 and 48 h for the uncontrolled and controlled nicotine, respectively, as depicted in Figure 4c. Just like the UV–Vis absorbance reading and equation (slope/intercept) = 0.4238/0667), respectively, were used to calculate the release fractions (Figure 7d). Similarly, the current generated and calibration curve data (Equation 2) were substituted in the formula to estimate the released fraction. In contrast, a lesser percentage for each interval was recorded. Overall, 83% and 80% cumulative release at 12 and 48 h for the bare/NIC and N‐SLNP were estimated (Figure 7a,b). Meanwhile, the preliminary screening of the delivery system (BF) showed no interference with nicotine detection within the potential window, as shown in Figure S3; hence, the converted generated current can be ideally predicted for the concentration of nicotine detected in the system. The fraction of NIC released from N‐SLNP was calculated using the following equation:

(5) $$\mathrm{Cumulative}\;\mathrm{drug}\;\mathrm{release}\;\%=\frac{Q_{t}}{Qv}\times 100$$

where $Q_{t}$ is the quantity of encapsulated nicotine released from the SLNP at that time *t*, whereas $Q_{v}$ is the quantity of NIC previously loaded into the BF‐SLNP.

### 3.5 Potential Applicability in Release Studies and Real Samples (Serum and Urine) Analysis

**FIGURE 8:**
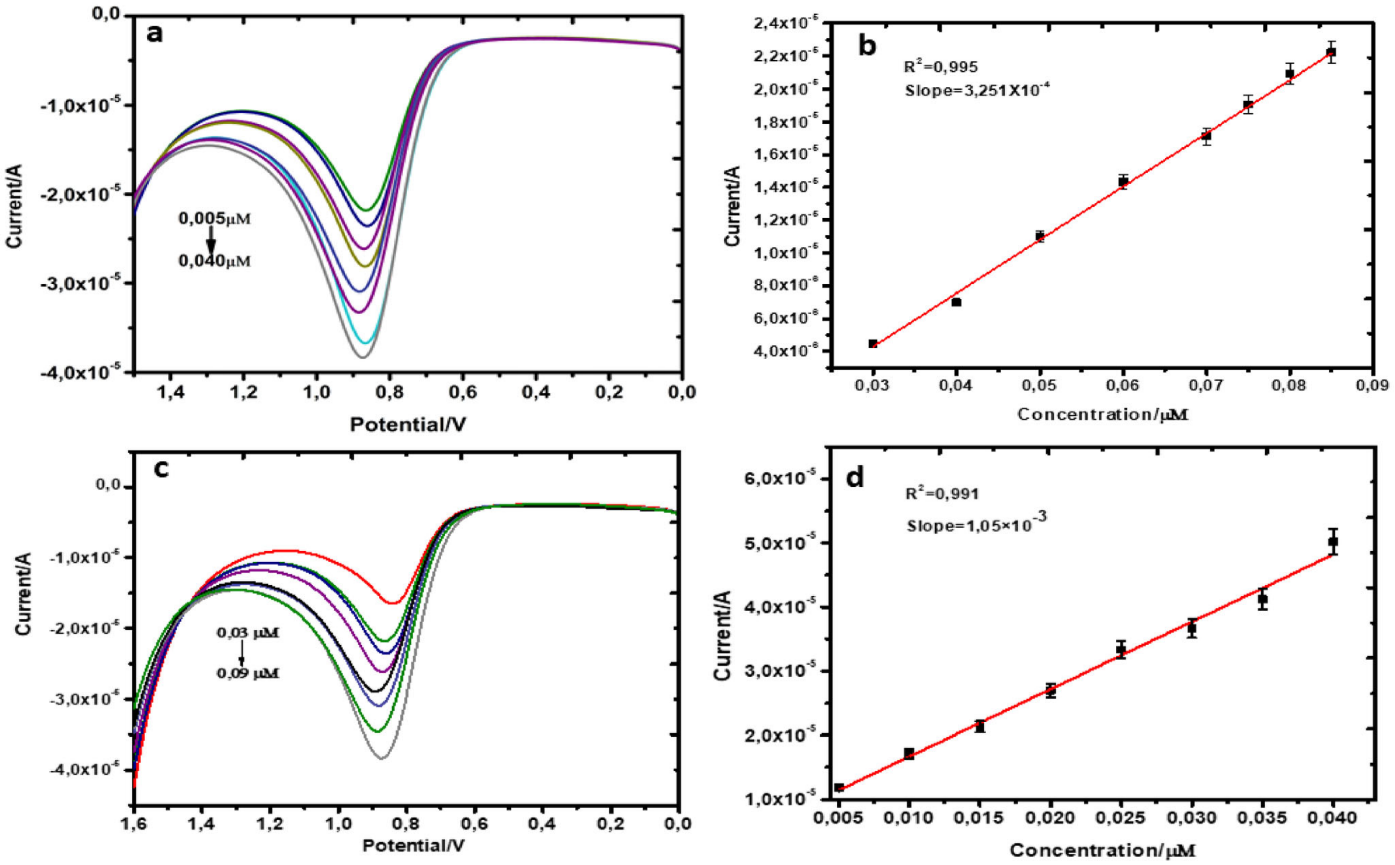
(a and c) Serum and urine and their respective linear plots (b and d) of N‐SLN.

Biological matrices, such as blood, urine and saliva, are crucial for drug testing and diagnosis because they are critical media for sample excretion. The matrix selection may be impacted by the kind of drug being tested for, the amount of time since administration, and the specifications of the testing methodology. These essential considerations led to selecting invasive (blood) and non‐invasive (urine) matrices, considering the administration routes/times and their detection windows [71]. The practicality of the modified electrode was assessed by diluting sheep serum 100 times. The sensor detected the formulated nicotine in urine and serum samples (Figure 8a,b). The graph created a linear regression equation for serum and urine, as expressed in the following equations:

(6) $$\begin{array}{cc} I_{P} & =3.251\times 10^{-4}\left(N-\mathrm{SLN}\right)\mu M-5.453\times 10^{-6}\left(R^{2}=0.995\right)\\ & \;\mathrm{Eqn}.5I_{p}=1.050\times 10^{-3}\left(N-\mathrm{SLN}\right)\mu M\\ & \;-6.180\times 10^{-6}\left(R^{2}=0.991\right) \end{array}$$

### 3.6 Interference, Reproducibility and Recovery Studies

**FIGURE 9:**
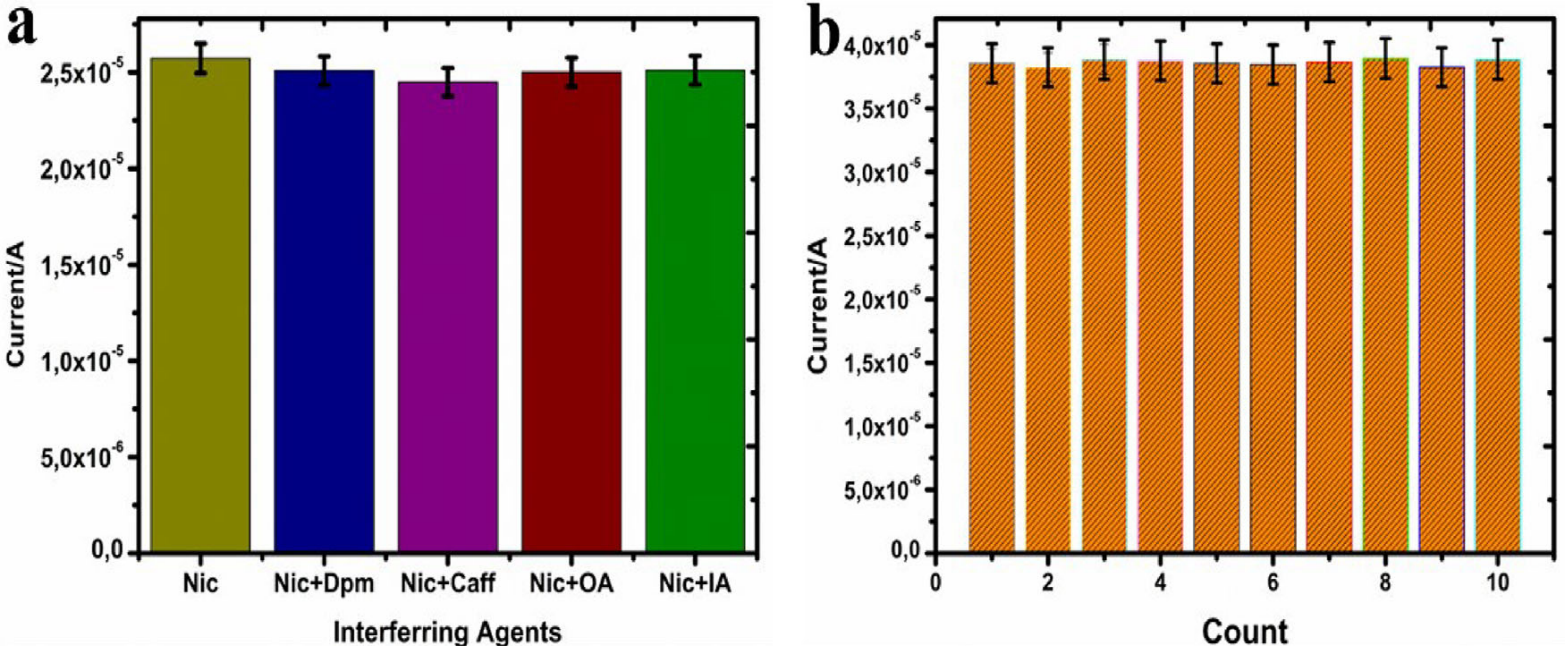
(a and b) Column graph of interference and reproducibility studies.

The sensor's selectivity for detecting the entrapped nicotine was tested in a mixture of organic (OA) and inorganic agents (IA) (glucose, ascorbic acid, uric and citric acids) and (Mg^2+^, K^+^, CO_3_, and SO_4_^2−^), respectively, as well as similar drug agents (dopamine and caffeine). The findings suggest the capacity of the sensor to sustain a significant sensitive and selective attraction towards nicotine. The results indicated no significant fluctuation in the *I*_p_ except for caffeine (Figure 9a). Meanwhile, caffeine showed a more substantial reduction in Ip (interference) but did not impede nicotine's electrochemical response and detection. The average peak was 2.50 × 10^−5^ A, with an relative standard devaition (RSD) of 4.6%, which is still within the 5% acceptable limit.

In addition, analyses of 10 independent identical sensor measurements were prepared to confirm the method's reproducibility, and the resulting outcome shows the method's repeatability (Figure 9b). All 10 measurements in the current study show that the electrode's performance is significantly reproducible, as the error bars indicate.

The practicability of the release studies and quantification of nicotine in the experimented doped urine and blood samples was assessed in a 100‐fold dilution. The recovery findings for the corresponding h in urine samples were analysed using DPV techniques, as presented in Table 3. The recovery values within the recommended ranges were 94%–98%, with an average recovery of 96.26%, as indicated in Table 3. Our study suggests that the electrochemical technique could be a reliable qualitative and quantitative technique for detecting and monitoring nicotine products and release.

**TABLE 3:** Recovery results for released nicotine solid nanoparticle (N‐SLNP).

Electrochemical quantification of urine spiked in vitro released sample			
H	Amt added (µM)	Amt found (µM)	%Recovery
**4**	10	9.69	96.9
**8**	15	14.30	95.3
**12**	20	18.99	94.9
**24**	25	24.03	96.1
**48**	50	49.07	98.1
**Average**			96.26
**RSD**			1.14

Abbreviation: RSD, relative standard devaition.

In addition to the significant average recovery rate and RSD, the electrochemical sensor developed in this study features several novel highlights. This includes the first reported conjugated nicotine electrochemical detection and release monitoring, a significant advancement in the field. In addition, the study's applicability to real samples, urine and serum shows potential for doping control, diagnostic, and quality control purposes. Thus, the impact of this research can be far‐reaching for an advanced analytical technique that can be applied to various fields beyond nicotine research, such as chemotherapeutic drug delivery and monitoring of nano‐formulated drugs.

## 4 Conclusion

The development of an electrochemical sensor for detecting and quantifying nicotine in various products is crucial for several informed reasons for public health implications, regulatory frameworks and addiction treatment. Nicotine addiction is a significant public health concern, with approximately 8 million deaths annually attributed to tobacco use. Accurate detection and quantification of nicotine levels in products can inform regulatory decisions and harm reduction as well as more effective addiction treatment. Thus, the study developed a novel electrochemical technique for detecting and monitoring both fast and sustained‐release nicotine for 48 h with a limit of detection and quantification of 2.283 × 10^−9^ and 0.761 × 10^−8^ M and an average recovery rate of 96.26% was successful. The electrochemical technique suggests a significant boost in detecting and monitoring controlled and uncontrolled nicotine products. This indicates the potential utility of this electrochemical sensor in clinical and biomedical, immigration points, in doping scenarios in sports, and even in crime scenes for monitoring slow‐release nicotine. The electrochemical method also showed improved sensitivity and specificity towards the analyte of interest. Overall, this study underscores the potential application of electrochemical sensing of nicotine in various forms and intended domains like environmental monitoring and public health, quality control and consumer safety and forensic science.

## Author Contributions

**Blessing Wisdom Ike**: conceptualisation, methodology, investigation, formal analysis, and writing the original draft. **Joshua C. Nwabuife**: delivery methodology, validation and review of the manuscript. **Lungelo Miya**: validation. **John Alake**: review of the manuscript and validation. **Darko Kwabena Adu**: review of the manuscript and validation. **Zondi Nate** and **Ruchika Chauhan**: review of the manuscript and validation. **Rajshekhar Karpoormath**: writing – review and editing, validation, supervision, software, resources, project administration, funding acquisition. **Mbuso Faya**: conceptualisation, review and editing, supervision, software, resources, project administration, funding acquisition.

## Conflicts of Interest

The authors declare no conflicts of interest.

## Supporting information



Supporting Information

## Data Availability

This published article and its Supporting Information section include all data generated or analysed during this study.
