# Supplementary material for: Release Monitoring and Detection of Formulated Solid Nanoparticle–Conjugated Nicotine in Blood and Urine Using Electrochemical Technique
Source: Anal Sci Adv. 2025 May 11;6(1):e70018. doi: 10.1002/ansa.70018 (PMC12066092; doi:10.1002/ansa.70018)
Supplement: Supplementary file 1 — Supporting Information [file ANSA-6-e70018-s001.docx]

**Supplementary Data**


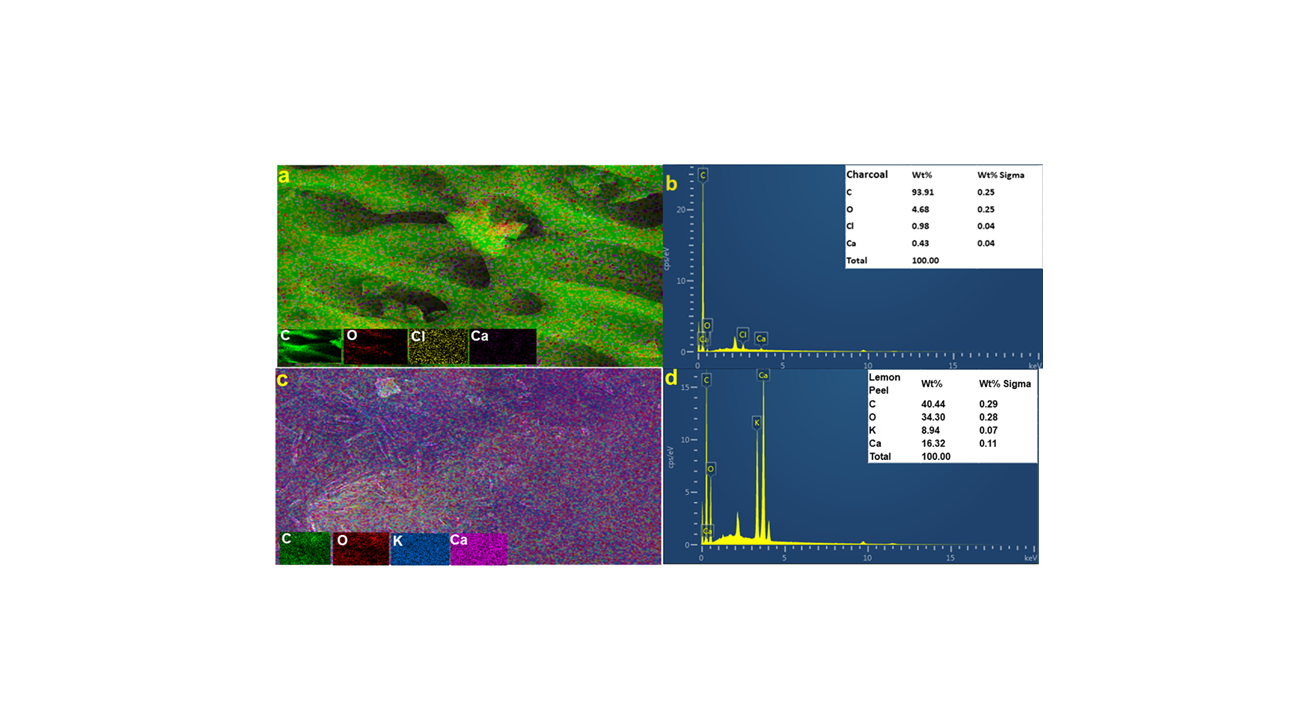
**Figure S1:** (a-B) Energy-dispersive X-ray mapping of Charcoal and (c-d) Energy-dispersive X-ray mapping of lemon peel and the corresponding spectrum, respectively.


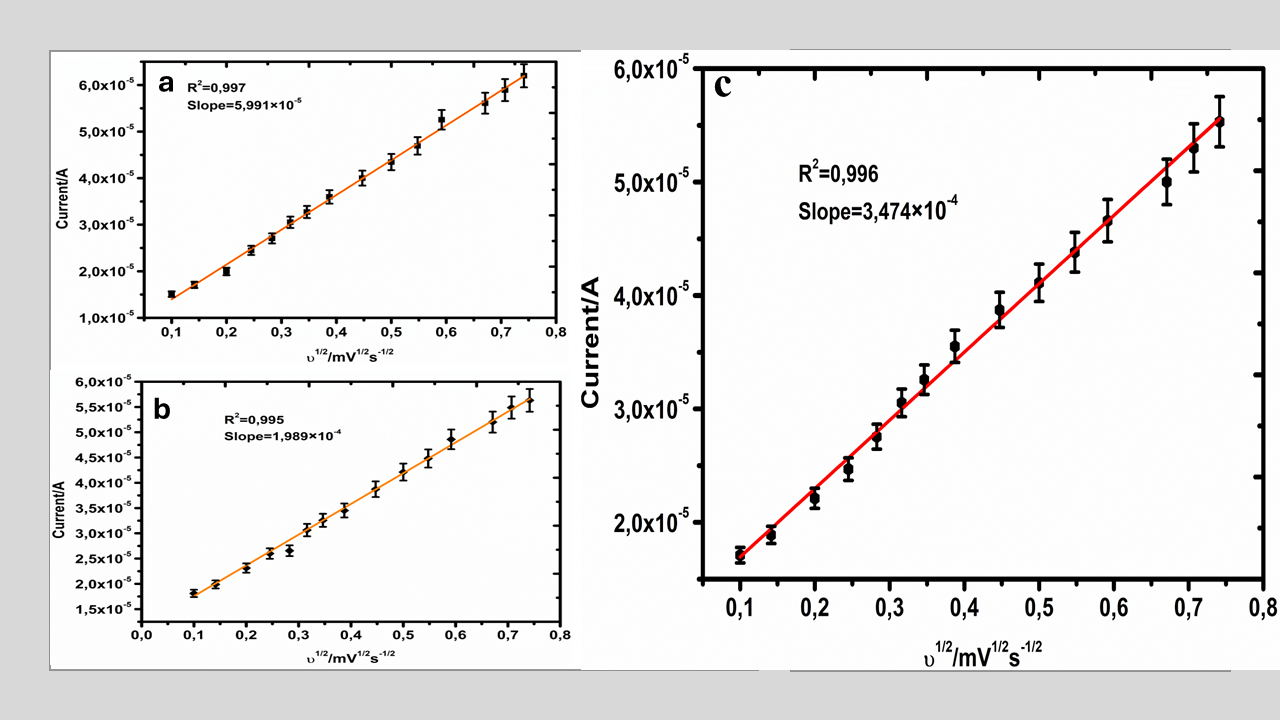


**Figure S2:** (a-c) Linear plots of scan rates for bare, char and AgNpC GCE, respectively.

**Figure S3:** Electrochemical performances of GCE
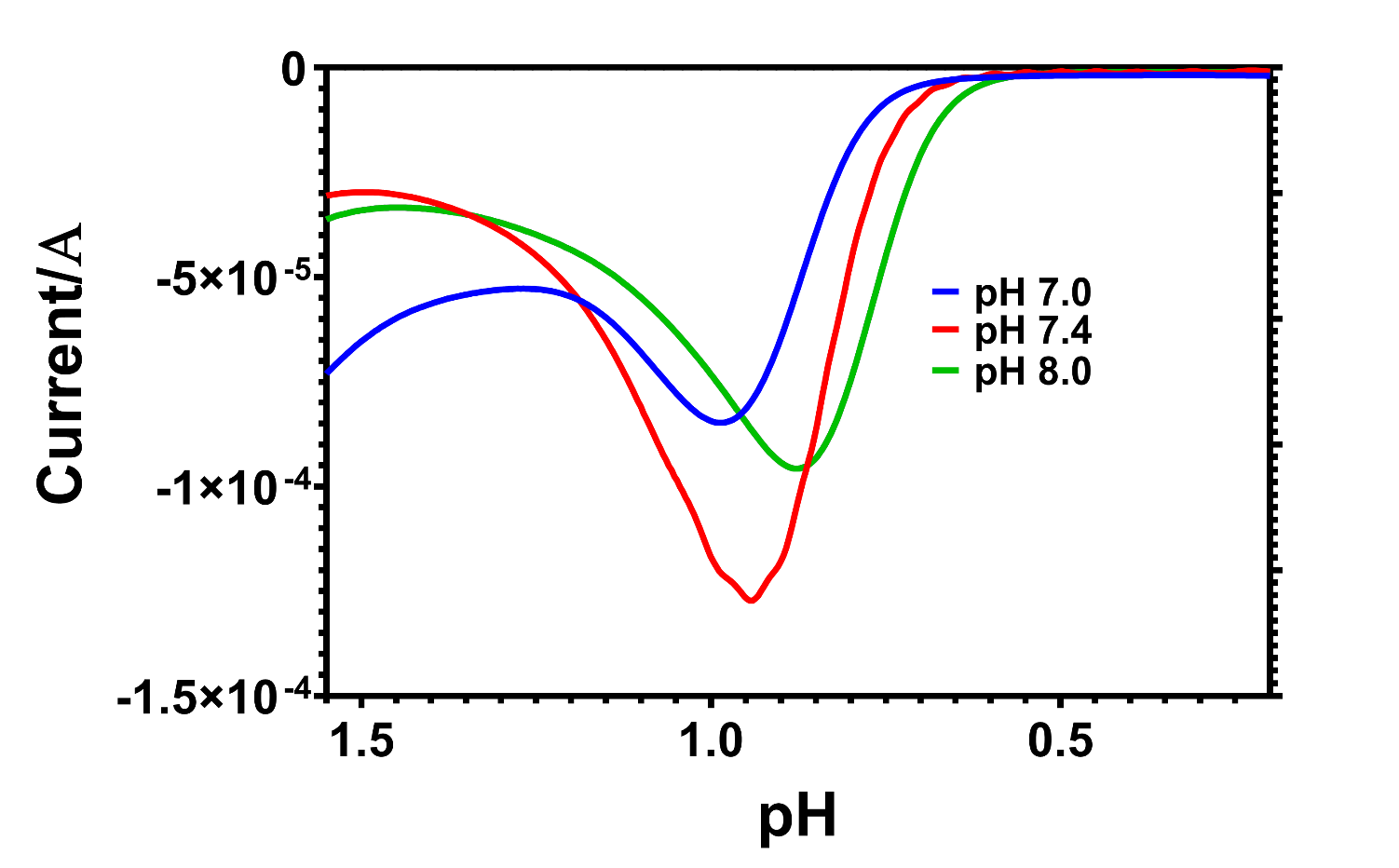
**

**

**Figure S4:** pH study
